# A Meta-Analysis of Local Adaptation in Plants

**DOI:** 10.1371/journal.pone.0004010

**Published:** 2008-12-23

**Authors:** Roosa Leimu, Markus Fischer

**Affiliations:** 1 Institute for Biochemistry and Biology, University of Potsdam, Potsdam, Germany; 2 Institute of Plant Sciences, University of Bern, Bern, Switzerland; 3 Section of Ecology, University of Turku, Turku, Finland; Oxford University, United Kingdom

## Abstract

Local adaptation is of fundamental importance in evolutionary, population, conservation, and global-change biology. The generality of local adaptation in plants and whether and how it is influenced by specific species, population and habitat characteristics have, however, not been quantitatively reviewed. Therefore, we examined published data on the outcomes of reciprocal transplant experiments using two approaches. We conducted a meta-analysis to compare the performance of local and foreign plants at all transplant sites. In addition, we analysed frequencies of pairs of plant origin to examine whether local plants perform better than foreign plants at both compared transplant sites. In both approaches, we also examined the effects of population size, and of the habitat and species characteristics that are predicted to affect local adaptation. We show that, overall, local plants performed significantly better than foreign plants at their site of origin: this was found to be the case in 71.0% of the studied sites. However, local plants performed better than foreign plants at both sites of a pair-wise comparison (strict definition of local adaption) only in 45.3% of the 1032 compared population pairs. Furthermore, we found local adaptation much more common for large plant populations (>1000 flowering individuals) than for small populations (<1000 flowering individuals) for which local adaptation was very rare. The degree of local adaptation was independent of plant life history, spatial or temporal habitat heterogeneity, and geographic scale. Our results suggest that local adaptation is less common in plant populations than generally assumed. Moreover, our findings reinforce the fundamental importance of population size for evolutionary theory. The clear role of population size for the ability to evolve local adaptation raises considerable doubt on the ability of small plant populations to cope with changing environments.

## Introduction

Local adaptation is of fundamental importance in evolutionary, population, conservation, and global-change biology. However, while it is commonly assumed that most plant populations are locally adapted the generality of local adaptation in plants and whether and how it is influenced by specific species, population and habitat characteristics is not clear. Currently, many plant populations are small and isolated and at the same time often facing rapidly changing environments to which they need to adapt to. The ability to adapt may, however, be compromised in small populations because of reduced genetic diversity [1]–[3]. Quantitative genetics theory predicts that the potential to respond to selection, and therefore also the potential to adapt decreases linearly with decreasing effective population size [4], [5]. Because effective population size depends on population dynamics, age structure and spatial population structure, it is generally closely related to, but nevertheless smaller than, census population size [6]. In contrast to the negative fitness effects of small population size mediated by reduced genetic diversity and increased inbreeding, which have been a major research focus [7], [8], and despite the fundamental role of population size in the early discussion on the evolution of adaptation between Fisher and Wright [9], the effects of population size have hardly been considered in studies on local adaptation in plants [but see 10], [11].

In addition to reduced genetic variation and genetic drift, local adaptation can also be constrained by variation in natural selection and gene flow [1], [12]. Temporal environmental variability may involve opposing selection pressures and thus constrain adaptation [13]. Moreover, temporal variability has been suggested to select for traits that increase propagule dispersal, which in turn also constrains local adaptation. In contrast, spatial heterogeneity of the habitats of plant origin favours selection for reduced dispersal and increases habitat fidelity [14], which may in turn favour the evolution of local adaptation. On the other hand, if local adaptation is constrained by lack of genetic variation, dispersal and gene flow between populations can enhance local adaptation by increasing genetic variation within populations and potential to respond to selection [15], [16]. It is generally assumed that the degree of local adaptation increases with increasing distance between populations, because of reduced gene flow among populations and the following increased genetic differentiation of populations [17], [18].

Plant traits such as mating system, longevity, and clonality have been suggested to affect the evolution of local adaptation mainly due to their effects on the level and distribution of genetic variation. Short-lived and self-compatible species tend to be more strongly differentiated at a smaller scale than long-lived and outcrossing species [19] and therefore the former are expected to show stronger adaptation to local conditions. Clonality can increase the potential for local adaptation if clonal growth restricts gene flow between habitats but allows morphological phenotypic plasticity via preferential placement of ramets within habitats [20]–[22]. Alternatively, clonal plants may be locally less adapted if long-lived genets are adapted to past conditions [23]. So far these hypotheses and their relative importance have not been examined in comparative studies involving different species and habitat types. Moreover, understanding the potential of plant populations to cope with anthropogenic environmental changes requires identifying the role of population size relative to other factors affecting local adaptation.

We conducted the first quantitative review on local adaptation in plants using 35 published studies on 32 plant species reporting 1032 pairwise comparisons of the performance of plants from local and foreign populations (See Table 1, and Supporting Material S1). We used these data for two approaches to examine the generality of local adaptation in plants, and the impact of population size, plant life history, temporal and spatial habitat heterogeneity, and geographic scale on the ability to adapt. As a first approach, we conducted a comprehensive meta-analysis [24] to examine whether local plants perform, on average, better than foreign plants at the site of origin and whether the average difference between local and foreign plants is influenced by species, habitat and study characteristics.

**Table 1:** Plant, study and habitat characteristics of studies included in the meta-analysis.

Species	Longevity	Mating system	Clonality	Type of exeriment	Habitat choice	Temporal constancy	Spatia hetero-geneity	Population size	Number of comparisons	Effect size (d)	Variance	Reference
*Arabidopsis thaliana*	Annual	SC	Non-Clonal	R	NR	C	HE	S	16	0.036	0.013	Callahan & Pigliucci 2002
*Gilia capitata*	Annual	SC	Non-Clonal	R	NR	NC	HO	L	2	0.276	0.196	Nagy 1997
*Potamogeton pectinatus*	Annual		Clonal	R	NR	C	HO		35	0.004	0.037	Santamaria et al. 2003
*Diodia teres*	Annual	SC	Non-Clonal	R	NR	NC	HE	L	12	−0.157	0.118	Hereford & Moriuchi 2005
*Arabis holboellii*	Perennial	SI	Clonal	R	RA	NC	HE	L	2	0.220	0.706	Roy 1998
*Bromus erectus*	Perennial	SI	Non-Clonal	E	RA	C	HO	L	4	−0.247	0.202	Ehlers & Thompson 2004
*Lupinus guadalupensis*	Annual	SC	Non-Clonal	R	NR	NC	HE	S	6	0.033	0.405	Helenurm 1998
*Ranunculus adoneus*	Perennial	SC	Non-Clonal	R	NR	C	HE	L	18	−0.207	0.058	Stanton & Galen 1997
*Arrhenatherum elatius*	Perennial	SC	Non-Clonal	E	NR	NC	HO	S	4	0.049	0.203	Petit & Thompson 1998
*Impatiens capensis*	Annual	SC	Non-Clonal	R	NR	NC	HO	L	8	−0.289	0.033	Schmitt & Gamble 1990
*Impatiens capensis*	Annual	SC	Non-Clonal	R	NR	C	HO	L	8	−0.379	0.120	Donohue et al. 2000
*Ranunculus reptans*	Perennial	SI	Clonal	E	NR	C	HE	S	12	0.033	0.322	Lenssen et al. 2004
*Diodia teres*	Annual	SC	Non-Clonal	R	NR			L	10	−1.174	0.250	Jordan 1992
*Lotus corniculatus*	Perennial	SI	Non-Clonal	E	NR	C	HO	L	3	−0.244	0.007	Smith et al. 2005
*Carlina vulgaris*	Perennial	SI	Non-Clonal	R	R	C	HO	S	68	−0.017	0.127	Jakobsson & Dinnetz 2005
*Hordeum spontaneum*	Annual	SC	Non-Clonal	R	NR	NC	HO		65	−0.682	0.447	Volis et al. 2002
*Gilia capitata*	Annual	SC	Non-Clonal	R	NR	NC	HO	L	14	−0.618	0.146	Nagy & Rice 1997
*Succisa pratensis*	Perennial	SC	Non-Clonal	E	RA	C	HO		10	0.640	0.244	Vergeer et al. 2004
*Hordeum spontaneum*	Annual	SC	Non-Clonal	R	NR	C	HE	L	8	−0.188	0.015	Verhoeven et al.2004
*Chamaecrista fasciculata*	Annual	SC	Non-Clonal	R	RA	NC	HE	L	12	−0.229	0.011	Etterson 2004
*Aristida stricta*	Perennial	SI	Non-Clonal	R	RA	C	HO	L	4	−0.285	0.077	Kindell et al. 1996
*Impatiens pallida*	Annual	SC	Non-Clonal	R	NR	C	HO	L	8	−0.060	−0.060	Bennington & McGraw 1995
*Hordeum spontaneum*	Annual	SC	Non-Clonal	R	NR	C	HO	L	7	−0.064	0.020	Volis et al. 2002
*Triplasis purpurea*	Annual	SC	Non-Clonal	E	NR	C	HO	S	4	0.179	0.187	Cheplick & White 2002
*Dialium guianense*	Perennial		Non-Clonal	R	NR	C	HO		2	0.179	0.101	Boege & Dirzo 2004
*Poa secunda*	Perennial	SC	Non-Clonal	R	NR	NC	HO	L	4	0.061	0.292	Link et al. 2003
*Zostera marina*	Perennial		Clonal	R	RA	NC	HO	L	4	−0.755	0.243	Hämmerli & Reusch 2002
*Hydrocotyle bonariensis*	Perennial	SI	Clonal	R	NR	NC	HO	L	23	−0.261	0.205	Knight & Miller 2004
*Anoda cristata*	Annual	SC	Non-Clonal	R	NR	C	HO	S	15	−0.096	0.140	Rendon &Nunez-Farfan 2000
*Spartina anglica*	Perennial		Clonal	R	NR	NC	HO	L	23	0.250	0.234	Thompson et al. 1991
*Anthoxanthum odoratum*	Perennial		Clonal	R	NR	NC	HO	L	12	0.006	0.072	Platenkamp 1990
*Ambrosia artemisiifolia*	Annual	SI	Clonal	R	NR	C	HO	S	10	−0.077	0.262	Genton et al. 2005
*Chamaecrista fasciculata*	Annual	SC	Non-Clonal	E	RA	NC	HO		71	0.087	0.226	Galloway & Fenster 2000
*Dactylis glomerata*	Perennial		Clonal	R	RA	C	HO	L	201	−0.280	0.211	Joshi et al. 2001
*Plantago lanceolata*	Perennial	SI	Non-Clonal	R	RA	C	HO	L	156	−0.272	0.220	Joshi et al. 2001
*Trifolium pratense*	Perennial	SI	Non-Clonal	R	RA	C	HO	L	159	−0.237	0.230	Joshi et al. 2001

SC = Self-compatible, SI = Self-incompatible, R = reciprocal transplant experiment, E = experimental test environments, RA = habitats/sites selected randomly, NR = sites selected because of clear habitat differences, NC = environments/habitats not constant in time, C = environments/habitats constant in time, HE = spatially heterogeneous habitats, HO = spatially homogeneous habitats, L = large populations (>1000 individuals), S = small populations (<1000 individuals), number of comparisons refers to local-foreign comparisons (i.e. individual effect sizes) per study and species.

**Figure 1:**
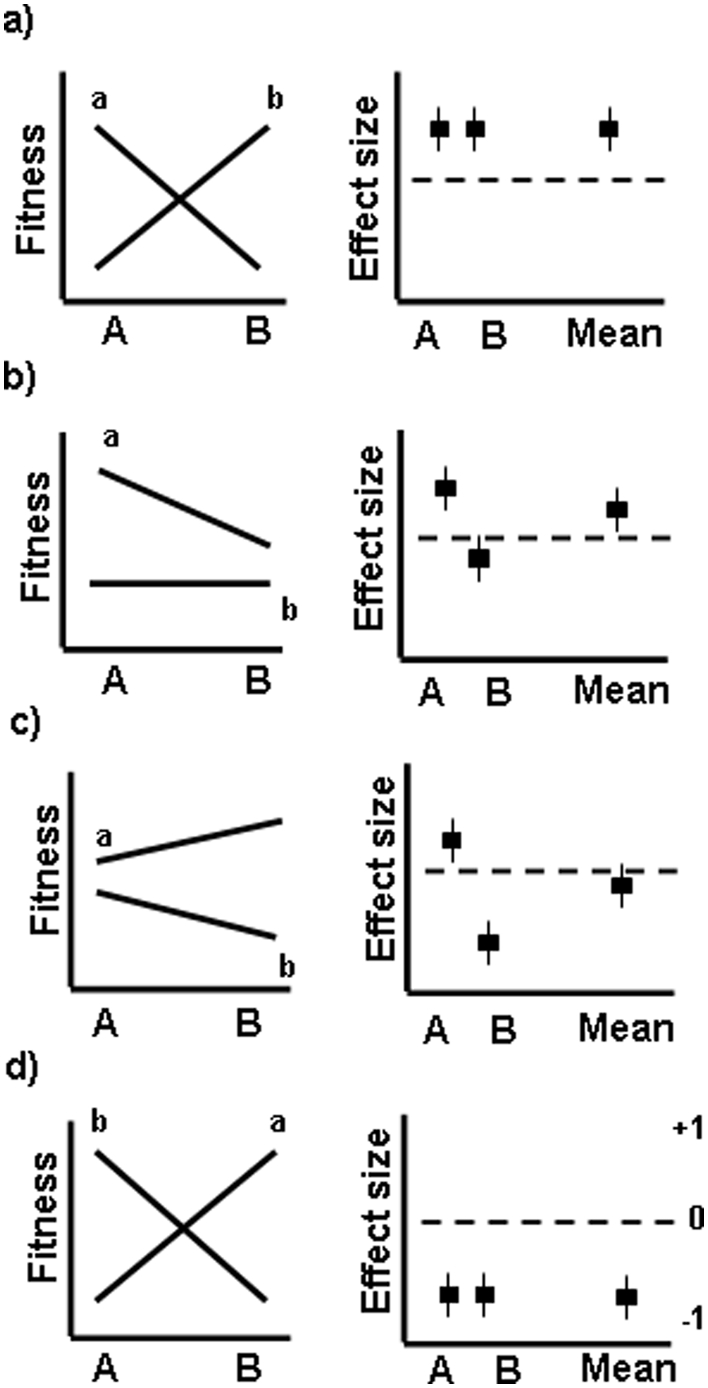
Conceptual graphs of the possible combinations of reaction norms for fitness and corresponding effect sizes (*Hedges' d*). The effect size measures the difference in fitness of foreign and local plants (“a” or “b”) at one site (“A” or “B”). A positive effect size indicates that local plants perform better than foreign plants at their site of origin. A) The case where local plants perform better than foreign plants at both compared sites, i.e. where the reaction norms for fitness cross and both effect sizes are positive ( = POS-POS). B, C) Plants of one origin (“A”) perform better at both compared sites. In this case of non-crossing reaction norms for fitness one effect size is positive and one is negative ( = POS-NEG). The resulting mean effect size can be positive (B) or negative (C). D) Foreign plants perform better than local plants at both sites indicating maladaptation (effect sizes negative = NEG-NEG).

However, in the strict sense (sensu Kawecki and Ebert [12]) examining local adaptation requires comparing the performance of local and foreign plants in a reciprocal manner between two sites or habitat. Therefore, we also took another approach in which we tested whether local plants perform better than foreign plants at both transplanting sites, i.e. pairs of plant origin, which would indicate divergent selection and thus more rigorous evidence for local adaptation [12]. Because the standard meta-analytical techniques do not allow such analysis we analyzed the frequencies of cases where the measures of plant performance were higher for local plants at both sites (“POS-POS”- case of crossing reaction norms, where both effect sizes are positive, Fig. 1a), at only one site (“POS-NEG”-case of non-crossing reaction norms, Fig. 1b, c), or at none of the two sites (“NEG-NEG”- case of crossing reaction norms, Fig. 1d).

## Results

### Generality of local adaptation in plants

Our meta-analysis revealed that, overall, local plants clearly outperformed foreign plants (Effect size Hedges' *d* = 0.1594, *N* = 36, 95% *CI* = 0.2499 to 0.0736). Moreover, among all individual comparisons of local and foreign plant origins at one site local plants performed better than foreign plants in 71.0% of the cases.

The reaction norms for fitness crossed (i.e. the respective local plants outperformed foreign ones in both compared environments; Fig 1) in 45.3% of the cases, indicating divergent selection and thus evidence for local adaptation sensu Kawecki and Ebert [12]. The high frequency (51.4%) of cases where the reaction norms did not cross and one population outperformed the other in both compared sites indicates, in turn, at least partial lack of local adaptation (the frequencies of the two cases “POS-POS” and “NEG-POS” did not differ: *χ^2^* = 0.521, *N* = 205, *P* = 0.471). Finally, maladaptation, indicated as better performance of foreign plants at both compared sites (“NEG-NEG”), occurred in only 3.3% of the cases (the frequency of this case differed from the other two cases: *χ^2^* = 88.22, *N* = 212, *P* = 0.0001).

### Population size and local adaptation

**Figure 2:**
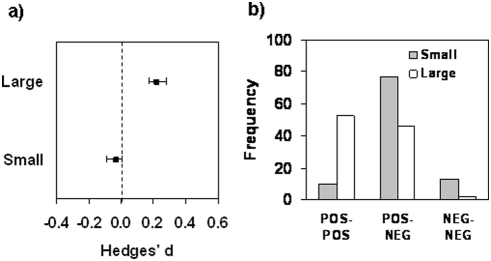
Relationship of plant population size and local adaptation. A) The better performance of local plants compared to foreign plants is significantly greater for large (N = 24) than for small (N = 8) populations. The bars denote bias-corrected 95% confidence limits. B) The frequencies of cases where reaction norms for fitness cross (POS-POS, see Fig. 1) indicating selection for locally adapted specialists, cases where the reaction norms do not cross (POS-NEG), and cases where effect sizes are negative at both sites indicating maladaptation (NEG-NEG). White bars denote large populations and grey bars denote small populations.

Local plants performed better than foreign plants in a given environment only in large plant populations as indicated by a significant positive overall effect size, whereas in small populations there was no significant difference in plant performance between plant origins (Fig. 2a; test for difference between large and small populations: *Q*_b_ = 5.50, *df* = 1, *N* = 32, *P* = 0.0026; this is smaller than the Bonferroni adjusted alpha level 0.0071). In addition, the frequencies of crossing and non-crossing reaction norms for fitness indicate that in large populations divergent selection and local adaptation sensu Kawecki and Ebert [12] (“POS-POS” case) occurred in 52.3% of the cases (Fig. 2b) whereas in small populations this was much rarer (9.3%, Fig. 2b). Furthermore, almost all maladapted pairs of populations were small (“NEG-NEG” case; Fig. 2b). These frequencies differed significantly between large and small populations (*χ^2^* = 25. 5, *N* = 212, *P* = 0.0001). These results suggest that small populations lack the potential to adapt to local environments.

### Plant life-history, habitat characteristics, geographic distance and local adaptation

**Figure 3:**
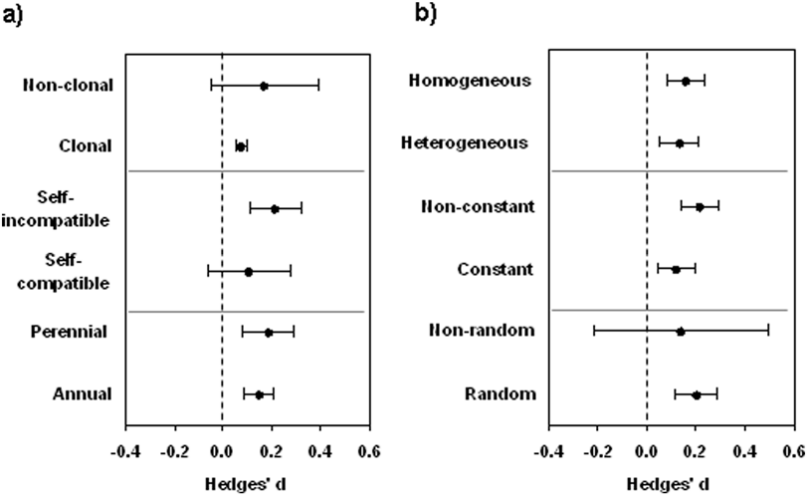
Evidence for local adaptation and effects of different plant and habitat characteristics. A) Effects of plant characteristics and B) of population characteristics on the effect size (Hedges' *d*). A positive effect size indicates better performance of local plants compared to foreign plants at a given site. Bars denote bias-corrected 95% confidence limits and the grey lines denote the pair-wise contrasts.

Local adaptation was independent of the plant or habitat characteristics considered in our study (Fig 3a, 3b). In our meta-analysis the strength and direction of the effect size did not differ between the considered categories of plant longevity (*Q*_b_ = 0.138, *df* = 1, *N* = 36, *P* = 0.755), mating system (*Q*_b_ = 1.666, *df* = 1, *N* = 31, *P* = 0.271), or clonality (*Q*_b_ = 0.528, *df* = 1, *N* = 36, *P* = 0.491) (Fig 3a). Moreover, the strength and direction of the effect size did not depend on the measure of temporal constancy of the habitats (*Q*_b_ = 0.051, *df* = 1, *P* = 0.784), on whether the sites had been selected randomly or because of specific habitat differences (*Q*_b_ = 0.122, *df* = 1, *N* = 36, *P* = 0.743), or on whether the habitats were considered spatially heterogeneous or homogeneous by the authors (*Q*_b_ = 0.213, *df* = 1, *N* = 36, *P* = 0.545) (Fig 3b). We found no difference in the strength and direction of the effect size between the reciprocal transplant studies and the experimental studies (*Q*_b_ = 0.245, *df* = 1, *N* = 36, *P* = 0.614; *d* = −0.14, *CI* −0.22 to −0.06 and *d* = −0.18, *CI* −0.23 to −0.24, respectively). Also, none of these descriptors of life-history or habitat characteristics was related to how frequently the reaction norms crossed.

**Figure 4:**
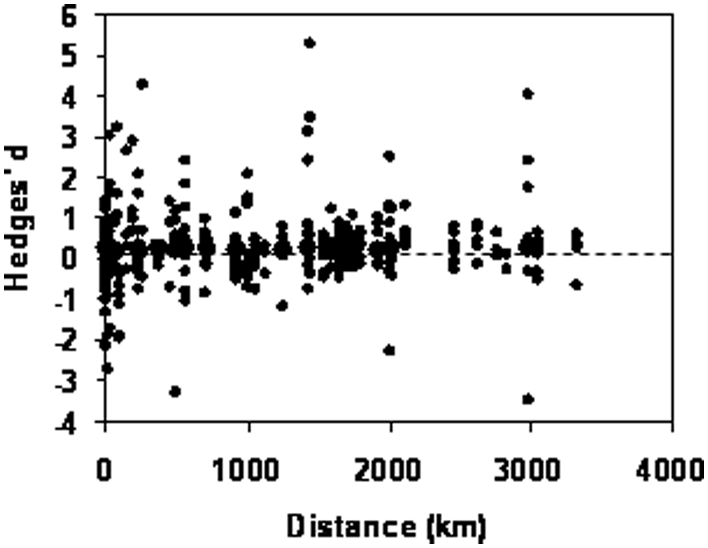
Relationship of geographic distance and local adaptation. For the graph we pooled the data for each pair of plant origins by the traits reported for this pair. For the statistical tests reported in the text we also used data pooled by study and species to avoid pseudoreplication.

The strength or direction of the effect size were not significantly associated with geographic distance between the compared sites of plant origin (Pair-wise comparisons of plant origins pooled by traits: *N* = 429, *Q*_b_ = 2.241, *df* = 1, *P* = 0.134, Fig 4; Pair-wise comparisons pooled by species and study: *N* = 26, *Q*_b_ = 0.133, *df* = 1, *P* = 0.715). However, variation in the strength of adaptation was greater at smaller than at larger geographic scale (Negative correlation between log-distance and residual effect size; *r* = −0.11, *N* = 429, *P* = 0.0235). This suggests that, although smaller-scale environmental variation was on average large enough to lead to local adaptation, this was less consistently so than at larger scales.

## Discussion

### Degree of local adaptation

Our meta-analysis reveals that on average, local plants perform better than foreign plants at their site of origin. Overall, this was found in 71.0% of the transplant sites. However, the pair-wise comparisons of the performances of local and foreign plants at both of the two sites between which plants had been reciprocally transplanted revealed that local plants performed better at both compared sites in only 45.3% of the cases. Only the latter finding gives accurate evidence of divergent selection and thus local adaptation in the strict sense suggested by Kawecki and Ebert [12]. These results highlight the importance of the definitions and conceptual issues raised by Kawecki and Ebert [12] for the study of local adaptation. In conclusion, while we clearly document evidence for local adaptation, it is less frequent than commonly assumed.

### Population size and local adaptation

A major finding of our study is that large plant populations are generally locally adapted whereas this is unusual for small populations. This is both indicated by the higher overall effect size for large populations and by the much higher frequency of POS-POS cases for pairs of large compared to pairs of small populations (Fig. 2). NEG-POS pairs can be interpreted as evidence for selection for generalists rather than for locally adapted specialists. Alternatively, even if NEG-POS would be interpreted as consisting of one adapted and one unadapted population, and therefore half of the NEG-POS pairs would be considered as evidence of local adaptation, there would still remain a much higher likelihood of local adaptation for large (76%) compared to small (49%) populations.

Using precise estimates of population sizes would have allowed us to analyze the effects of population size on adaptation in a more detailed way and, for example, to examine potential threshold population sizes for adaptation. Unfortunately, due to temporal and demographic variation it is difficult to accurately estimate population sizes in the field. Therefore, for our study the authors could only provide the very coarse “small” or “large” estimates of population size. However, although these estimates are coarse there is no reason to believe that they would have biased our results. On the contrary, more precise estimates might even have resulted in a closer relationship between population size and local adaptation.

Small populations can have a low evolutionary potential and fail to adapt locally for various reasons. Firstly, larger populations can accumulate higher levels of heritable variability and beneficial mutations and might therefore respond to selection better than small populations do [5], [25]. Secondly, local adaptation may be masked by high inbreeding depression in small populations. Thirdly, if populations remain small for a long time period, or if population bottlenecks occur, drift can lead to the loss even of advantageous alleles [26]. Fourthly, the observed maladaptation in small populations can be due to genetic drift linked to founder effects, especially if the founders of the small populations originate from contrasting environments [27].

### Independence of local adaptation of species, habitat, and study characteristics

Plant responses to environmental variation may depend on plant life history. However, our study did not confirm any prediction on the roles of species longevity, mating system and clonality for local adaptation (Fig. 3a). Moreover, how commonly this was true for both compared sites (crossing reaction-norms, Fig. 1) or not (non-crossing reaction norms) was not influenced by any of these traits. It is likely that a larger number of studies and more precise definitions of these life-history characteristics might lead to the detection of such effects in future meta-analyses. However, our study strongly suggests that the magnitude of the effect of these factors is much smaller than the one of the effect of population size.

Local plants performed better than foreign plants regardless of whether plant origins had been selected randomly by the experimenters or based on clear differences in the compared environments and regardless of whether plants were transplanted reciprocally in the field or to deliberately designed test environments. This matters from a methodological point of view, as it excludes the possibility that the studies used in our meta-analysis could have been biased towards pronounced local adaptation due to selection of study systems. In addition, it suggests that local adaptation is not necessarily driven by obvious environmental differences between habitats. Of course these considerations only hold to the degree to which the experimenters were able to identify the environmental factors relevant for adaptation.

We detected no effects of the spatial or of the temporal heterogeneity of the compared habitats on local adaptation. Thus, our study suggests that effects of temporal and spatial heterogeneity on the evolution of local adaptation are either less important than previously thought, or that they cancel each other. Nevertheless, it must be kept in mind that the authors could only provide quite coarse estimates of spatial and temporal heterogeneity. Therefore, these considerations only hold to the degree that seemingly homogeneous habitats do not actually provide heterogeneous conditions for the organism under study, as found in some studies [28], [29]. Possibly, when more studies reporting the necessary data will become available, increased statistical power could allow us separating between alternative explanations. This would also allow us to more precisely estimate the means and confidence intervals of effect sizes for the different categories of species or habitat characteristics. In addition, long-term experiments manipulating the spatial and temporal heterogeneity of otherwise equivalent habitats would be very helpful. Nevertheless, even if more detailed studies will reveal significant effects of temporal or spatial heterogeneity on local adaptation, our study strongly suggests that the magnitude of these effects will be much smaller than the one of the effect of population size.

### Independence of local adaptation of geographic distance

It has been suggested that the likelihood of detecting local adaptation increases with greater geographic distance between compared sites, because genetic isolation and environmental differences usually increase with increasing distance [17]. In contrast, although we examined a geographic range spanning six orders of magnitude, from 3 meters to 3500 km, we found no effect of distance on the strength of local adaptation (Fig. 4). Clearly, the effect of geographic distance on local adaptation depends on the association of geographic distance with the actual environmental differences acting as selective agents. For example, in previous large-scale reciprocal transplantation experiments including plant origins from different climates in N and S Europe the performance of transplants decreased with distance from the home site [30]. Nevertheless, the general independence of local adaptation from geographic scale among the 32 studies supports the idea that the average magnitude of environmental variation is generally comparable at small and large geographic scales [17]. Alternatively, adaptation may not be increased in long-distance comparisons once the distance is greater than the scale of local adaptation [31]. However, we found the same average degree of local adaptation for all distances.

While, as just discussed, the mean level of local adaptation was independent of the distance between the sites, variation in the strength of local adaptation was greater at smaller than at larger geographic scale. This indicates that environmental conditions are always very likely to differ between geographically distant populations, whereas the conditions between populations that are geographically less distant apart can either be similar or very different.

### Conclusion

To date, studies on local adaptation in plants are only available for herbaceous plants in temperate regions. While among these studies local genotypes performed on average better than foreign genotypes at their site of origin, selection favoured locally adapted plants only in less than half of the pair-wise site comparisons. This suggests that local adaptation is less widespread than commonly believed. In contrast to a wealth of hypotheses brought up during recent decades, local adaptation appeared to be independent of the considered plant life-history traits, the degree of spatial and temporal habitat heterogeneity, and of the geographic distance between study populations. In contrast to all other tested factors potentially affecting local adaptation studied in our meta-analysis population size had a very large and clear effect. The much lower likelihood of local adaptation in small populations reinforces the fundamental interest of population size for evolutionary theory. In addition, the clear role of population size for the evolution of local adaptation raises considerable doubt on the ability of small plant populations to cope with changing environments.

## Materials and Methods

### Data acquisition and meta-analysis

The standard method to examine local adaptation in plants is the reciprocal transplant experiment, where plants from different populations are either transplanted between these field populations or to corresponding test environments. The latter refers to experiments where for instance plants from a dry and from a wet meadow are planted both to dry and wet experimental environments. To search for such studies we conducted key word searches in the Web of Science (ISI) database using combinations of the key word “plant” with “local adaptation”, “reciprocal transplant*”, “adaptation” and “adaptive evolution”. The search resulted in a list of 211 articles. Moreover, we screened the reference lists of these articles to identify further potentially relevant articles. The criterion for including published studies in our meta-analysis was that they reported mean values, variance and sample sizes of performance of local plants and foreign plants at one or several sites. Because early studies on local adaptation [e.g. 32]–[35] either did not reciprocally transplant between sites, did not report the data required for meta-analysis, or because it was not possible to inquire further information from the authors, they could not be included. The final data set consisted of data from 35 articles on 32 plant species (Table 1, Supporting Material S1). Twenty-eight of these articles reported results of reciprocal transplant experiments in the field and seven of experiments where plants of different origin had been transplanted to test environments that represented environmental differences observed in the field.

The reported fitness-relevant measures included measures of plant reproductive success (such as the number of fruits, flowers, or seeds, fruit set, or seed set), plant size (such as biomass, leaf size, plant height, or number of ramets), survival rates, and germination rates. We extracted altogether 1032 pairwise comparisons of the performance of local plants and foreign plants at a given site. For each performance measure we calculated the effect size, Hedge's *d* [36], as the difference between the means of local plants compared to foreign plants in one environment divided by their pooled standard deviation and multiplied by a correction term to account for a bias caused by small sample size [24]. A positive effect size indicates greater performance of the local plants compared to foreign plants in a given environment.

To test for the importance of different sources of variation we classified our data according to characteristics of populations, habitats, studies, and plant life-history. This data was to a large extent obtained directly from the authors. As population characteristic we tested the effect of population size. Information on population size had been provided only in very few articles. Thus, we inquired this information directly from the authors. Since population size had not been considered explicitly in most of the studies, the authors could not provide count data on population sizes but were able to state with certainty whether populations in their experiments were smaller or larger than 1000 flowering individuals. Although a finer classification would have been desirable, we consider this coarse classification nevertheless appropriate, because genetic problems in terms of reduced genetic variation and increased inbreeding of small population have been predicted for population sizes lower than 100–1000 [e.g. 37], [38] and because a census population size of 1000 flowering individuals can be assumed to correspond to an effective population of even lower size [6], [39]. According to the authors the environments of their study populations had not changed shortly before the experiments, i.e. there is no reason to believe that the small populations were small due to recent changes in land use or other environmental factors. We also asked the authors to classify the habitats of plant origin either as spatially rather heterogeneous or rather homogeneous, and as temporally constant or inconstant. A more accurate classification of spatial and temporal habitat heterogeneity was not possible, because the authors had usually not considered these factors directly. Study characteristics were the type of experiment (reciprocal transplantation or transplantation to test environment) and whether the study sites had been selected randomly or deliberately according to obvious differences in habitat quality. Plant life-history traits included mating system (self-compatible or self-incompatible), longevity (annual or perennial), and clonality (clonal or non-clonal).

We avoided non-independent pair-wise comparisons, which would correspond to pseudo-replication, by pooling data by species and by measure of plant performance (reproduction, growth, survival, germination) when testing for effects of population size, life-history traits, type of study and habitat heterogeneity and homogeneity on the strength of the effect. Data was pooled by pair-wise site comparison to test for the effect of geographic distance on the strength of the effect (see below). When effect sizes for several measures of plant performance were obtained per study we pooled the data by calculating mean effect sizes and their pooled variances. Of course, it would be best to analyse whole life-cycle estimates of fitness rather than the single or few components provided by the published studies. At least, to some degree the pooling of data by study and species takes aspects of total fitness into account, because fitness components with opposing trends cancel out each other in pooling.

To test whether the effect sizes differed depending on the different plant, habitat or study characteristics we examined between-group heterogeneity using the chi-square test statistic, *Q_b_*. To account for the problem caused by multiple statistical tests we used the Bonferroni adjustment to modify the significance criterion (*α/k* where *k* = the number of statistical tests) [40]. In our case, p-values lower than 0.0071 can be considered statistically significant.

To examine the association of geographic distance and effect size we used random-effects continuous-model meta-analysis [41] weighting the effect sizes by the inverse of sampling variance. In addition, to examine whether variation in effect size was larger at smaller geographic scale, we calculated absolute values of residual effect size after fitting log-transformed geographic distance, and then tested whether these residuals were related to log-transformed geographic distance.

We used Meta Win 2.0 [41] to carry out mixed-model meta-analyses [24]. We calculated bias-corrected 95% bootstrap confidence intervals generated from 4999 iterations [42]. We considered an effect size significant if its confidence interval did not include zero. The funnel plot technique [43], [44] did not reveal any significant evidence for publication bias. Furthermore, effect size was not correlated with sample size (r = 0.046, P = 0.79) further supporting lack of publication bias. This also implies that sample sizes were not smaller for studies of smaller populations (see results).

### Analysis of types of reaction norms

We analysed the frequencies of cases where the measures of plant performance were higher for local plants at both sites (“POS-POS”- case of crossing reaction norms, where both effects sizes are positive, Fig. 1a), at only one site (“POS-NEG”-case of non-crossing reaction norms, Fig. 1b, c), or at none of the two sites (“NEG-NEG”- case of crossing reaction norms, Fig. 1d). We conducted maximum likelihood analyses of variance to test for the effects of the study, habitat, population, and plant life-history traits on the frequencies of the POS-POS and POS-NEG cases. For these analyses we excluded the NEG-NEG due to their low frequency of only 3.3%. The different fitness measures were pooled for the analysis in a similar manner as for the meta-analysis.

## Supporting Information

Supporting Material S1 List of studies included in the meta-analysis
(0.04 MB DOC)
