# Supplementary material for: A Meta-Analysis of Local Adaptation in Plants
Source: PLoS One. 2008 Dec 23;3(12):e4010. doi: 10.1371/journal.pone.0004010 (PMC2602971; doi:10.1371/journal.pone.0004010)
Supplement: Supporting Material S1 — List of studies included in the meta-analysis (0.04 MB DOC) [file pone.0004010.s001.doc]

**Supporting material:**

**Studies included in the meta-analysis:**

Bennington, C. C. and McGraw, J. B. 1995. Natural Selection and ecotypic differentiation in *Impatiens pallida*. Ecological Monographs 65:303-324.

Callahan, H.S. and Pigliucci, M. 2002. [Shade-induced plasticity and its ecological significance in wild populations of *Arabidopsis thaliana*](http://wos23.isiknowledge.com:80/?SID=Z6MEJ99k3gOECBofFF3&Func=Abstract&doc=2/103). Ecology 83:1965-1980.

Cheplick, G.P. and White, T.P. 2002. Saltwater spray as an agent of natural selection: No evidence of local adaptation within a coastal population of *Triplasis purpurea* (Poaceae). American Journal of Botany 89:623-631.

Donohue, K., Messiqua, D., Pyle, E.H., Heschel, M. S., & Schmitt, J. 2000. Evidence of adaptive divergence in plasticity: Density- and site-dependent selection on shade-avoidance responses in *Impatiens capensis*. Evolution 54:1956-1968.

Ehlers, B.K. and Thompson, J. 2004. Do co-occurring plant species adapt to one another? The response of *Bromus erectus* to the presence of different *Thymus vulgaris* chemotypes. Oecologia 141:511-518.

Etterson, J.R. 2004. Evolutionary potential of *Chamaecrista fasciculata* in relation to climate change. 1. Clinal patterns of selection along an environmental gradient in the great plains. Evolution 58:1446-1458.

Galloway, L.F. and Fenster, C.B. 2000. Population differentiation in an annual legume: Local adaptation. Evolution 54:1173-1181.

Griffith, C., Kim, E. and Donohue, K. 2004. Life-history variation and adaptation in the historically mobile plant *Arabidopsis thaliana* (Brassicaceae) in North America. American Journal of Botany 91:837-849.

Helenurm, K. 1998. Outplanting and differential source population success in *Lupinus guadalupensis*. Conservation Biology 12:118-127.

Hereford, J. and Moriuchi, K.S. 2005. Variation among populations of *Diodia teres* (Rubiaceae) in environmental maternal effects. Journal of Evolutionary Biology. 18:124-131.

Jakobsson, A. and Dinnetz, P. 2005. Local adaptation and the effects of isolation and population size - the semelparous perennial *Carlina vulgaris* as a study case. Evolutionary Ecology 19:449-466.

Joshi, J., Schmid, B., Caldeira, M.C.Dimitrakopoulos, P. G., Good, J. Harris, R., Hector, A., Huss-Danell, K., Jumpponen, A., Minns, A., Mulder, C. P. H., Pereira, J. S., Prinz, A., Scherer-Lorenzen, M., Siamantziouras, A.-S. D., Terry, A. C., Troumbis, A. Y. and Lawton, J. H. 2001. Local adaptation enhances performance of common plant species. Ecology Letters 4:536-544.

Lenssen, J.P.M., Van Kleunen, M., Fischer, M. & de Kroon, H. 2004. Local adaptation of the clonal plant *Ranunculus reptans* to flooding along a small-scale gradient. Journal of Ecology 92:696-706.

Nagy, E.S. and Rice, K.J. 1997. Local adaptation in two subspecies of an annual plant: Implications for migration and gene flow. Evolution 51:1079-1089.

Nagy, E.S. 1997. Selection for native characters in hybrids between two locally adapted plant subspecies. Evolution 51:1469-1480.

Petit, C. and Thompson, J.D. 1998. Phenotypic selection and population differentiation in relation to habitat heterogeneity in *Arrhenatherum elatius* (Poaceae). Journal of Ecology 86:829-840.

Roy, B.A.1998. Differentiating the effects of origin and frequency in reciprocal transplant experiments used to test negative frequency-dependent selection hypotheses. Oecologia 115:73-83.

Santamaria, L., Figuerola, J., Pilon, J.J., Mjelde, M., Green, A. J., de Boer, T., King, R. A. & Gornall, R. J. 2003. Plant performance across latitude: The role of plasticity and local adaptation in an aquatic plant. Ecology 84:2454-2461.

Schmitt, J. and Gamble, S. E. 1990. The effect of distance from the parental site on offspring performance and inbreeding depression in *Impatiensis capensis*—a test of the local adaptation hypothesis. Evolution 44:2022-2030.

Smith, B.M., Diaz, A., Winder, L., & Daniels, R. 2005. The effect of provenance on the establishment and performance of *Lotus corniculatus* L. in a re-creation environment. Biological Conservation 125:37-46.

Stanton, M.L. and Galen, C. 1997. Life on the edge: Adaptation versus environmentally mediated gene flow in the snow buttercup, *Ranunculus adoneus*. American Naturalist 150:143-178.

Vergeer, P., Sonderen, E., Ouborg, N.J. 2004. Introduction strategies put to the test: Local adaptation versus heterosis. Conservation Biology 18:812-821.

Verhoeven, K.J.F., Vanhala, T.K., Biere, A, Nevo, E. & van Damme, J. M. M. 2004. The genetic basis of adaptive population differentiation: A quantitative trait locus analysis of fitness traits in two wild barley populations from contrasting habitats. Evolution 58:270-283.

Volis, S., Mendlinger, S., and Ward, D. 2002. Differentiation in populations of *Hordeum spontaneum* Koch along a gradient of environmental productivity and predictability: plasticity in response to water and nutrient stress. Biological Journal of the Linnean Society 75:301-312.

Volis, S., Mendlinger, S., Ward, D. 2002. Adaptive traits of wild barley plants of Mediterranean and desert origin. Oecologia 133:131-138.

Volis, S., Mendlinger, S. and Ward, D. 2002. Differentiation in populations of *Hordeum* *spontaneum* along a gradient of environmental productivity and predictability: life history and local adaptation. Biological Journal of the Linnean Society 77:479-490.
